# Molecular evolution of rDNA in early diverging Metazoa: First comparative analysis and phylogenetic application of complete SSU rRNA secondary structures in Porifera

**DOI:** 10.1186/1471-2148-8-69

**Published:** 2008-02-27

**Authors:** Oliver Voigt, Dirk Erpenbeck, Gert Wörheide

**Affiliations:** 1 Dept. of Geobiology, Geoscience Centre Göttingen, University of Göttingen, D-37077 Göttingen, Germany

## Abstract

**Background:**

The cytoplasmic ribosomal small subunit (SSU, 18S) ribosomal RNA (rRNA) is the most frequently-used gene for molecular phylogenetic studies. However, information regarding its secondary structure is neglected in most phylogenetic analyses. Incorporation of this information is essential in order to apply specific rRNA evolutionary models to overcome the problem of co-evolution of paired sites, which violates the basic assumption of the independent evolution of sites made by most phylogenetic methods. Information about secondary structure also supports the process of aligning rRNA sequences across taxa. Both aspects have been shown to increase the accuracy of phylogenetic reconstructions within various taxa.

Here, we explore SSU rRNA secondary structures from the three extant classes of Phylum Porifera (Grant, 1836), a pivotal, but largely unresolved taxon of early branching Metazoa. This is the first phylogenetic study of poriferan SSU rRNA data to date that includes detailed comparative secondary structure information for all three sponge classes.

**Results:**

We found base compositional and structural differences in SSU rRNA among Demospongiae, Hexactinellida (glass sponges) and Calcarea (calcareous sponges). We showed that analyses of primary rRNA sequences, including secondary structure-specific evolutionary models, in combination with reconstruction of the evolution of unusual structural features, reveal a substantial amount of additional information. Of special note was the finding that the gene tree topologies of marine haplosclerid demosponges, which are inconsistent with the current morphology-based classification, are supported by our reconstructed evolution of secondary structure features. Therefore, these features can provide alternative support for sequence-based topologies and give insights into the evolution of the molecule itself. To encourage and facilitate the application of rRNA models in phylogenetics of early metazoans, we present 52 SSU rRNA secondary structures over the taxonomic range of Porifera in a database, along with some basic tools for relevant format-conversion.

**Conclusion:**

We demonstrated that sophisticated secondary structure analyses can increase the potential phylogenetic information of already available rDNA sequences currently accessible in databases and conclude that the importance of SSU rRNA secondary structure information for phylogenetic reconstruction is still generally underestimated, at least among certain early branching metazoans.

## Background

Tens of thousands of sequences of the small subunit ribosomal RNA (SSU rRNA, 18S) gene of eukaryotes have accumulated in public databases such as NCBI GenBank [1], making this gene one of the first and most frequently used markers for molecular phylogenetics. Its popularity is due to a high degree of conservation in some regions of the molecule, in combination with a considerable amount of variability in others. These features enable phylogenetic questions to be addressed between relatively closely related taxa, as well as between different domains of life [2]. Therefore, analyses of SSU rRNA sequences have a long history, and new sequences are still being continuously generated.

SSU rRNA molecules fold into a specific secondary structure, which is essential for maintenance of their three dimensional structure and their function within the ribosome [3], but which also has consequences for the use of rRNA molecules in phylogenetics. The secondary structure of rRNAs is maintained by hydrogen bonds between RNA nucleotides, which form helices (or stems). These helices are interleaved by regions consisting of unpaired nucleotides, forming loops at the end of a helix and bulges within different helices. Secondary structure of RNAs is generally much more conserved than their primary sequence [2]. Therefore, considering this structure during multiple sequence alignment can greatly improve the assignment of homologous positions, consequently resulting in more probable phylogeny estimations [4-6]. Furthermore, paired nucleotides (= doublets) frequently co-evolve in order to maintain rRNA structure and function. The co-evolution of doublets violates the assumption of independent evolution of sites made by most phylogenetic methods [7]. Consequently, specific evolutionary models have been proposed for paired sites and have been shown to outperform standard (4 × 4) nucleotide models [8-14]. However, secondary structure models are still rarely used in phylogenetic analyses, presumably because establishing a secondary structure for a new sequence is still a time-consuming exercise even for the conserved core structure of SSU rRNA, and very few software packages allow the simultaneous analysis of paired and unpaired rRNA regions. Some rRNA databases [15-18] provide secondary structure information for a number of organisms, but their records are far from complete and structures of hypervariable insertions are usually not presented, or are only presented to a certain extent. In particular, the lower Metazoa, which are pivotal for the understanding of animal evolution, are still under-represented in databases.

One key taxon for early metazoan evolution is Phylum Porifera (sponges), in which the relationships are unresolved at all taxonomical levels, even between the three extant sponge classes Demospongiae, Calcarea (calcareous sponges) and Hexactinellida (glass sponges). Within sponge classes, the results of molecular phylogenies are often incongruent with morphological expectations [13,19-22]. In this study, we performed the first comprehensive survey of the complete SSU rRNA secondary structures of representatives of the main lineages of Phylum Porifera, and evaluated how secondary structure information and features other than the primary sequence can contribute to improve phylogenetic reconstructions. For these purposes, we considered all available SSU rRNA sequences of Porifera, inferred their secondary structures (a selection of which we are presenting in a new database), and analyzed base compositions and sequence lengths. We reconstructed a phylogeny with partitioned phylogenetic analyses using specific rRNA models of nucleotide evolution for paired sites. Using this backbone, we assessed the phylogenetic value of secondary structures of unique insertions found in a specific demosponge clade (Order Haplosclerida), which would usually be disregarded as 'unalignable sites' and thus excluded from standard phylogenetic analyses.

## Methods

### Sequence acquisition, analyses and inference of secondary structures

We analyzed all 170 published full or nearly full-length SSU rRNA sequences of Porifera (see Additional file 1 for a complete listing). For taxonomy of the taxa included in our study we followed Systema Porifera [23] and the World Porifera Database [24], where also the species authorities are available. The SSU rRNA sequence of *Amphimedon queenslandica *was reconstructed by performing a local Blast search [25] against data from GenBank's trace archive. Traces from significant hits (see Additional file 2) were downloaded and assembled in CodonCode Aligner 1.6.3 [26]. This resultant sequence can be downloaded from our database of SSU rRNA secondary structures of Porifera [27]. For Class Hexactinellida, only limited data was available in GenBank: All three full-length SSU rRNA sequences belong to Subclass Hexasterophora. Two additional hexactinellid sequences were provided by Martin Dohrmann ahead of their publication in a comprehensive phylogenetic study of Hexactinellida [28]: *Semperella schulzei *(subclass Amphidiscophora) and *Aphrocallistes vastus *(Subclass Hexasterophora).

All sequences were initially aligned with CLUSTAL W 1.83 [29] and the preliminary alignments were manually improved in SeaView [30]. Gblocks 0.91b [31] was used to identify and isolate the conserved sites of the alignment before clustering similar sequences using the Neighbor Joining (NJ) algorithm in PAUP* 4.0b10 [32]. Secondary structures for resulting clades were established for certain representatives of the clade by aligning to known structures from the European RNA Database [18,33] in separate alignments for each clade and considering compensatory base changes. SSU rRNA clade-alignments were then further refined according to secondary structure information.

The unusual structures of marine Haplosclerida (= Order Haplosclerida excluding Suborder Spongillina) and Hexactinellida (including conserved flanking regions with known structure) were initially examined under minimum free energy predictions from the mfold-server [34]. In most cases, only one structure was predicted by the algorithm. If multiple structures were predicted, we chose the structure with either the minimal free energy or with the best compatibility to similar sequences.

A comparative approach [35] was chosen if permitted by an appropriate level of sequence divergence. For this approach, we used the alifold server [36] to infer secondary structures of the insertions. Alifold infers secondary structures by considering both minimum free folding algorithms and compensatory base changes, and therefore includes additional information that provides hints for secondary structural motifs. Since this method requires a correct alignment, it could only be used if sequences were not too divergent from each other, such as with a subset of marine Haplosclerida (Demospongiae) and the insertions of Hexasterophora (Hexactinellida) (Additional file 3). However, secondary structures inferred with both methods were identical, or only differed in a few positions (Additional file 3, p. III). Therefore, while the comparative method is preferred, we still found that minimum free energy based predictions performed adequately to be used in cases where unambiguous alignments or missing comparative data does not allow inference of secondary structures based on compensatory base exchanges. For taxa that were suitable for a comparative approach, compensatory base exchanges are presented together with the corresponding alignments in Additional file 3.

We visualized selected structures by converting the sequence and structure information to a ct-format with a Perl-script. This format can be displayed in RNAviz 2 [37,38]. Helix names correspond to Wuyts et al. [39], with the exception of helices E23_1 and E23_2, which together are referred to as E23_1. Insertions are designated by the name of the conserved helix in which they occur, and a period plus the number of the additional helix is added: Parts of conserved helices separated by insertions are named after the original helix followed by a letter (e.g., one helical insertion within E23_1 will be called E23_1.1, the 5' part of the helix before the insertion will be called E23_1a, the 3' part after the insertion E23_1b).

Base compositions and the lengths of the secondary structure features were calculated with a custom-made Perl script. To avoid biases introduced by missing data from the published sequences, we used a fragment (corresponding to ca. 95% of SSU rRNA) spanning from helix 5 until 2 bp before helix 50 (i.e. positions 48–1896 in *Amphimedon queenslandica*), and only considered the 123 sequences without data missing within this region (listed in Additional file 4). Representative poriferan secondary structures are available as *.fasta-format (with bracket-dot annotation) and in *.ct-format from our database for SSU rRNA secondary structures of Porifera [40]. Furthermore, several Perl scripts (written for Mac OS X/Linux) for format conversion are provided (along with other scripts: Tools for conversion from annotated alignments to ct-format and vice versa, and from alignments to MrBayes or PHASE data-files containing the secondary structure information are included).

### Phylogenetic analyses

The secondary structure information from the previous step was used to generate a new alignment in SeaView. We generated a taxon-set comprising of 78 taxa (for accession numbers see Additional file 5) and focused on relationships of haplosclerid demosponges, in a similar way to Redmond et al. [41]. The SSU rRNAs from this diverse taxon have been found to possess numerous insertions and extensions and our aim was to unravel their evolution.

Sites with uncertain homology even after considering secondary structure were excluded from the phylogenetic analyses. This was achieved by assigning sites to two groups and discarding those sites that were regarded as ambiguously aligned by the following criteria:

1. Unpaired sites: with length polymorphism and sequence divergence too high to identify homologous positions for all sequences. (Bulge after 3' helix 8; loops of helices 6, 10, E10_1,11,17, E23_12, 29, 44, 49)

2. Paired sites: with length polymorphisms in helices and/or structural homologies that could not be unambiguously assigned (e.g., in cases of elongation of helices, parts of helices 10, E10_1, E23_1/E23_2, 49).

Furthermore, taxon-specific insertions within helices (found in some marine Haplosclerida), as well as nucleotide insertions found only in single sequences were excluded.

Doublet positions were only regarded as pairings in the consensus secondary structure if the two involved nucleotides formed a Watson-Crick (G-C, A-U) or G-U wobble pairing in at least five sequences within the alignment. Corresponding sites falling below this five-sequence threshold were treated as unpaired. For phylogenetic reconstructions, sites were allocated to one of the following two partitions: Partition 'stem' (= paired sites) or partition 'loop' (= unpaired sites). We used MrBayes 3.1.2 [42] and PHASE 2.0 [43] for the phylogenetic analyses, as both programs allow the simultaneous analysis of a partitioned dataset with both rRNA models for paired sites and standard models for unpaired sites.

MrBayes only allows the usage of a doublet model corresponding to the SH model [9]. This is a 16 state-RNA model, which considers all possible doublets as characters and assumes that compensatory base exchanges result from at least two substitution events. A GTR + G + I model [44] was assigned for the loop partition. The Markov chain Monte Carlo (MCMC) analysis comprised two runs (eight chains each) for 12.142 million generations, with the sample frequency set to 100 and the temperature for the heated chains set to 0.2. Sampled trees were summarized using the *sumt *command in MrBayes with a burn-in set to the first 2 million generations. Sufficient convergence of chains for the Mr Bayes runs was monitored by observing log-likelihood values, the standard derivation of split frequencies (> 0.008), and diagnostics provided by AWTY [45,46].

In PHASE, we applied the RNA7A model [2] and RNA7D model [11] for stem regions in independent runs. RNA7A is the most general 7-state RNA model. RNA7D (seven frequencies, four rate parameters) is a simplification of RNA7A (7 frequencies, 21 rate parameters). The 7-state RNA models treat all mismatches as one single state. This simplification increases the risk of loss of phylogenetic information, but the occurrence of mismatch-pairs in rRNA data is small, therefore, an estimation of mismatch substitution parameters from the data is probably not accurate [47]. Furthermore, by pooling mismatches into a single character, the number of parameters to be estimated in a phylogenetic analysis, and consequently the computational demands are significantly decreased. For loop regions, the REV model [44] was chosen. In addition, a gamma distribution accounting for rate heterogeneity among sites and a proportion of invariant sites were assigned to each model for both partitions. Independent runs were performed in PHASE 2: Two runs with the RNA7A model (40 million generations) and one run (5 million generations) with the RNA7D model for stem positions. Every 100th generation a sample was taken from the MCMC chains (after a burn-in-phase of 1 million generations).

Tracer v1.4 [48] was used to monitor sufficient parameter stabilization. To create readable input files for Tracer from the PHASE runs, we used a slightly modified version of the perl script 'phase2tracer.pl' (originally programmed by Matt Yoder [49]), which is available upon request.

The presented tree topology is based upon one of the 40 million PHASE runs with the RNA7A model for stem partition (loop model as mentioned above). To obtain branch-lengths for the tree, we conducted an additional analysis (4 million generations) under the same models, and tree topology was fixed to the consensus tree from the original 40 million generation analysis as suggested in the PHASE manual (all other parameters unchanged).

## Results

### SSU rRNA length differences and base composition

**Figure 1:**
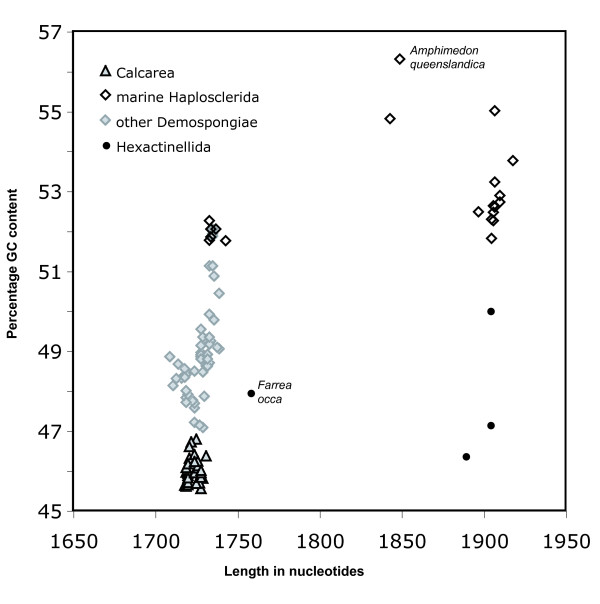
**GC content against SSU rRNA fragment length**. (Fragment corresponds to *A. queenslandica *positions 48–1896). A ca. 95% -fragment of SSU rRNA was used for analysis and only sequences with sequence information over the whole range of this fragment were considered (n = 123). Note that *Farrea occa *(Hexactinellida, [GenBank: AF159623]) is an incomplete potential pseudogene sequence.

To avoid biases due to missing data, we analyzed base composition and sequence length for a fragment of SSU rRNA that covers about 95 % of the gene (see Methods). Base composition and fragment length differed considerably among the 123 poriferan sequences (Fig. 1). The GC content varied between 45.5 and 56.3 %.

Calcarea posses the lowest GC contents with a modest variation from 45.5 to 46.8 %. In this aspect they are clearly separated from demosponges, which display significantly higher GC contents, since the lowest demosponge value (47.1%) still exceeds the highest GC content (46.8%) of Calcarea (Fig. 1). Most demosponge SSU rRNAs show modest length variations in a range comparable to those of Calcarea. Notable exceptions are the extraordinary large rRNA molecules found in several marine haplosclerids. The highest GC contents of Porifera are also found within this group (with a maximum of 56.3% in *Amphimedon queenslandica*). The high GC pattern is independent of the presence of insertions in these large molecules, since members of marine Haplosclerida with smaller rRNA molecules also possess similar GC contents (Fig. 1). SSU rRNAs of the few available hexactinellid sponges are approximately equal in length to large molecules of several haplosclerids (with the exception of *Farrea occa *[GenBank: AF159623], see below). In contrast to haplosclerids, hexactinellid sponges have lower GC contents, with base compositions in the range of those of Demospongiae and Calcarea.

### Secondary structure

**Figure 2:**
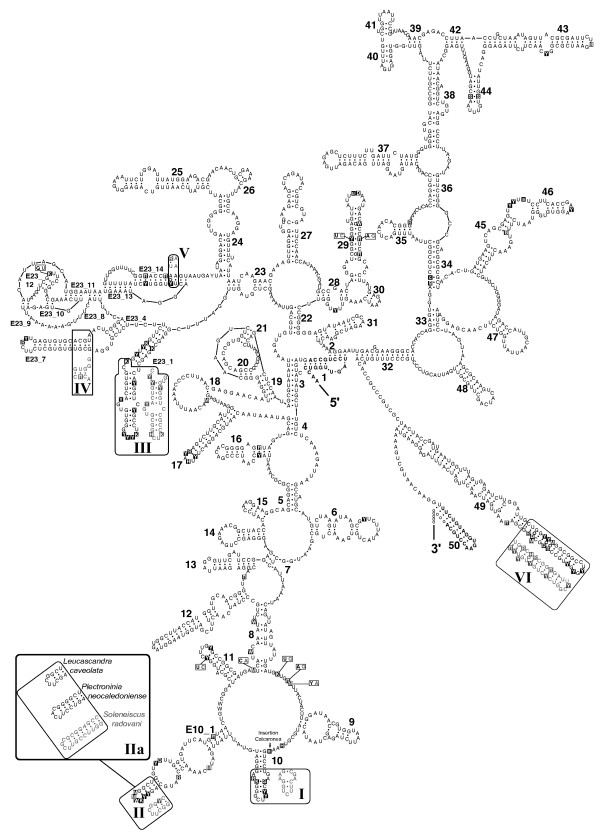
**SSU rRNA secondary structure for Calcarea**. Sequence is given as 90% consensus with variable positions in black boxes. Lower case indicates deletions at the site for some sequences, according to the consensus level. Differences in helices between Calcaronea and Calcinea are in frames (Calcaronea = black, Calcinea = grey). Synapomorphies for each subclass are shown in boxes with the same color code. Primer positions are bold at the 5' and 3' end, respectively. Open circles instead of dots mark positions where mismatches occur in some sequences. Inset: Shortening and elongations in the boxed part of Helix E10_1 for two calcaronean sequences and one calcinean sequence.

**Figure 3:**
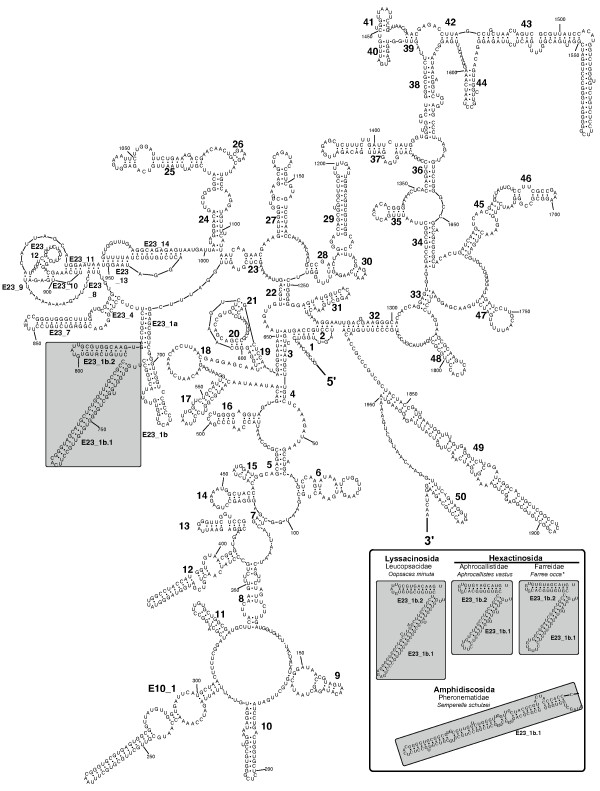
**SSU rRNA secondary structure of *Acanthascus dawsoni *[GenBank: **AF100949**] (Lyssacinosida, Rossellidae)**. Hexactinellid-specific helical insertions within E23_1 are shown in a box. Inset: Prediction of secondary structure insertions in E23_1 within other Hexactinellida. The insertions are predicted to form two helices in Hexasterophora (Lyssacinosida + Hexactinosida), and one helix in Amphidiscophora (*Semperella schulzei*). *Note that *Farrea occa *(AF159623) represents an (in other than the displayed part) incomplete, potential pseudogene molecule.

**Figure 4:**
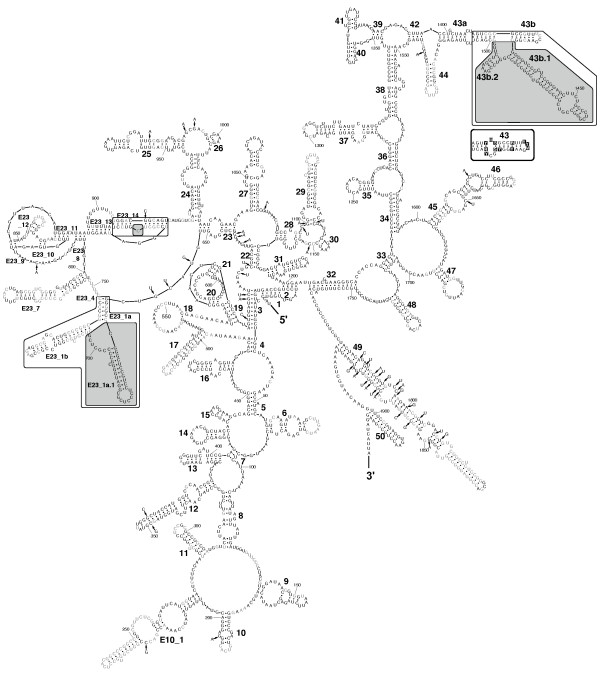
**SSU rRNA secondary structure of the demosponge *Amphimedon queenslandica *(Haplosclerida)**. Nucleotides conserved in Demospongiae at the 90% level are shown in black, other nucleotides are in grey. Nucleotides at positions that are present in demosponges above the 90% consensus level but differ from *A. queenslandica *nucleotides are shown with an arrow pointing to their corresponding position. Specific insertions for *A. queenslandica *that are atypical for demosponges are displayed in shaded frames. Outlined frames highlight the regions of insertion within Haplosclerida that are displayed as sketches in Fig. 6. Inset: 90% consensus sequence and structure of partial helix 43 for 76 demosponges that do not belong to the marine haplosclerids.

Porifera have the typical eukaryotic core SSU rRNA structure (see Figs. 2, 3, 4). The moderate length variation between Calcarea and most demosponges is primarily caused by insertions in unpaired regions or by elongation of helices 10, E10_1 and 43 (Table 1). In Hexactinellida, on average, these three helices are largely elongated compared to Calcarea and Demospongiae (Fig. 3), but the lengths of the E10_1 helices of some demosponge sequences fall into the same range.

**Table 1:** Mean and range of the length of the most variable helices within the three sponge classes

	**Calcarea **(n = 48)	**Demospongiae **(n = 109*)	**Hexactinellida **(n = 5**)
Helix	bp (range)	bp (range)	bp (range)
**10**	22.8 (21–26)	21.9 (20–30)	36.0 (35–37)
**E10_1**	55.4 (54–64)	61.6 (55–74)	75.4 (67–80)
**43**	49.1 (49–50)	49.5 (48–55)	91.5 (72–101)

*Marine haplosclerids not included in Helix 43; **Farrea occa not included for helix 43.

In addition, we observed extra-helical insertions in Hexactinellida and in several marine haplosclerid demosponges that are not part of the eukaryote core structure. In marine haplosclerids, these extra sequences were inserted within helices E23_1, E23_14 and 43, and in Hexactinellida, the insertions only occurred within helix E23_1 at a different position than in marine haplosclerids (Figs. 3, 4). All of the helices where sequence elongations and/or insertions occur belong to regions that are known to be highly variable within eukaryotes [39,50].

#### Calcarea

The SSU rRNA of this sponge class comprises all of the typical eukaryote helices and lacks unusual structural features. A calcarean SSU rRNA consensus sequence and structure is shown in Fig. 2. Several synapomorphies for the two Calcinea and Calcaronea subclasses were detected in the secondary structure. In Calcinea, helices 10 and E23_1 are shorter by at least one base pair when compared to Calcaronea (Fig. 2, insets I & III). In helix E10_1, Calcaronea typically have three pairs at the helix end, whereas Calcaronea dominantly possess four pairs (Fig. 2, inset II). However, independent elongations of this helix can be found in both subclasses (Fig. 2, inset IIa: Calcaronea: *Plectroninia neocaledoniense*; Calcinea: *Soleneiscus radovani*). These elongations are homoplasies as is evident when considering the subclass-specific compensatory base change (Calcaronea: A-U; Calcinea: G-C) at the beginning of inset II (Fig. 2): The A-U pair in the corresponding structure of the calcaronean *Leucascandra caveolata *(Borojevic & Klautau, 2000) supports a secondary loss of a pair compared to other Calcaronea. Differences in helix nucleotides between both subclasses occur in helices 11, E23_7, E23_14 and 29 (Fig. 2, and insets IV and V). Most of these changes maintain the helix-relevant pairings (e.g., in 11 or E23_7), but a few cause mismatches in at least some sequences (in E23_7, E23_14, 29 and 49). Base changes and insertions in unpaired regions are also specific for the Calcinea-Calcaronea split. This is indicated in Fig. 2 for three bases in the bulge between helices 8 and 9, one base within the loop of E23_12, and a calcaronean-specific insertion of one adenosine between helix 9 and 10.

#### Hexactinellida

The SSU secondary structure of *Acanthascus dawsoni *is presented in Fig. 3 representatively for Hexactinellida. In all hexactinellid sequences, specific insertions were observed (Fig. 3 inset). As mentioned previously, hexactinellid SSU rRNA sequences are considerably longer than in other poriferans, except in some marine haplosclerid demosponges (Fig. 1). The additional nucleotides occur in extensive elongations of common helices (10, E10_1, and 43; Table 1, Fig. 3), and a helical insertion in helix E23_1. The insertions in helix E23_1 occur at a unique position among sponges and may form two helices (assigned the names E23_1b.1 and E23_1b.2 in Fig. 3) in all the studied SSU rRNA molecules of subclass Hexasterophora. Helix E23_1b.2 contains 10 doublets and is much more conserved within Hexasterophora than Helix E23_1b.1, which varies in length from 37 to 55 bp. In contrast to Hexasterophora, *Semperella schulzei *(Subclass Amphidiscophora) has a helical insertion of 107 bp within E23_1, which is predicted to form a single helix E23_1b.1 (Fig. 3 inset).

Within the sequence of *Farrea occa *[GenBank: AF159623], we found deletions in conserved regions. Helices 13 and 15 are missing completely, as are the 3' strand of helix 7, parts of helix 43 and the 5' strand of helix 15 (compare Fig. 3). Such complete or partial deletion of conserved helices has been shown to be typical for non-functional rRNA pseudogenes [51]. Potential paralogs like this one are not necessarily subject to concerted evolution, and are therefore not suitable for phylogenetic inference. In this context, the consideration of secondary structure is crucial for identification of such non-functional sequences, and prevents biases in phylogenetic reconstruction due to potentially misleading data. Nonetheless, predictions of insertions for this sequence are displayed in Fig. 3 (inset), since no suspicious modifications were found within this part of the molecule and no other sequence of Farreidae was available. However, the results for this species should be treated with caution.

#### Demospongiae

**Figure 5:**
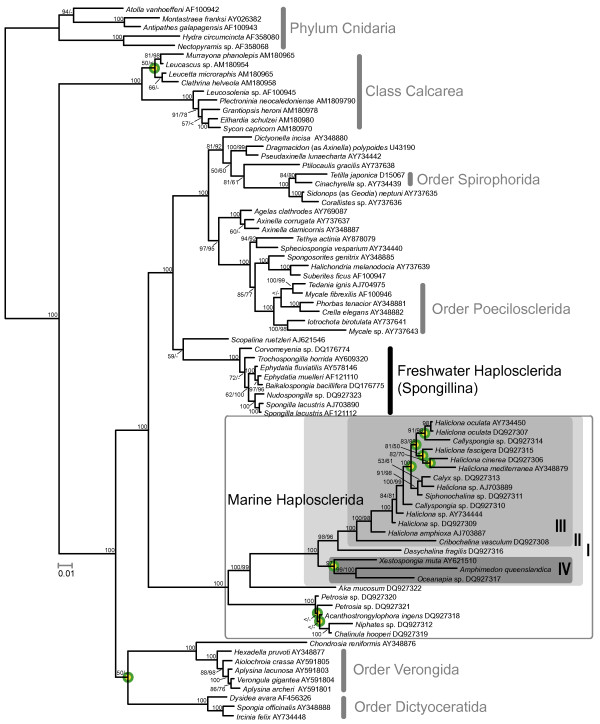
**Phylogeny inferred with PHASE**. Nodes that differ from the topology published by Redmond et al. [41] are encircled. The boxed clades correspond to the excerpt displayed in Fig. 6. Support values are given at, or close to the corresponding node (values from analyses with PHASE/MrBayes; where the same support values were found in both analyses, only one number is shown; '<' = support values below 50; '-' = node not recovered in MrBayes analysis.). Monophyletic higher taxa are assigned.

**Figure 6:**
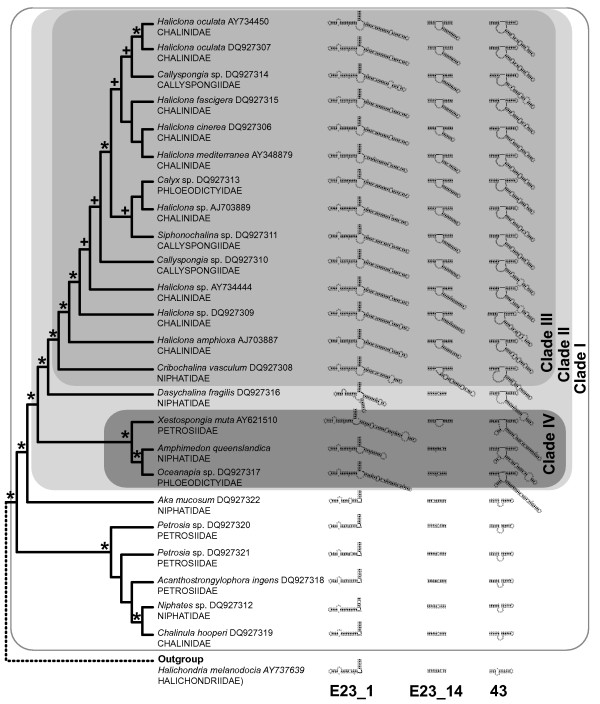
**Relationships of marine Haplosclerida (excerpt from larger phylogenetic analyses shown in Fig. 5) and evolution of extension regions**. Sketches of predicted secondary structures for extensions and conserved flanking regions correspond to outlined boxes in Fig. 4. Asterisks mark nodes that were found in at least 96% of sampled trees after burn-in in both Bayesian analyses (PHASE and MrBayes, see Material and Methods for details); plus signs mark nodes that appeared in lower frequencies, but still above 84% in one, or both of the analyses. For each species, the family is shown below the sequence name.

Most demosponges possess a SSU rRNA molecule with the common metazoan secondary structure. Remarkable exceptions are only found within the marine Haplosclerida (Figs. 4, 5, 6), which possess insertions that are long enough to be predicted to form additional helices. Those helices are found within known variable regions for eukaryotes and appear in the 5' strand of Helix E23_1/2, the 5'strand within Helix E23_14 and the 3' strand of helix 43.

### Phylogenetic analyses

We inferred the phylogeny of marine haplosclerids to compare the evolutionary history of helical insertions found in this group of Demospongiae (see section "Successive evolution of additional helices in marine haplosclerids"). Results from the PHASE- and MrBayes analyses of 78 taxa are shown in Fig. 5. Although more general 7-state models have been shown to result in higher likelihood values for phylogenies than less parameter-rich models for real rRNA data [47], our analyses with PHASE with the RNA7A model and the less complex RNA7D model yielded identical tree topologies (with almost identical support values). Independent runs in PHASE and MrBayes resulted in similar, almost identical topologies, and differences in demosponge relationships were only observed in the positions of clades with weak support values. Namely these are the relationship of Dictyoceratida to the Myxospongiae (sensu Borchiellini et al. 2004 [= clade Verongida +*Chondrosia reniformis*]), the position of *Scopalina ruetzleri *and relationships within freshwater sponges (where branch lengths were short, Fig. 5). Additionally, differences were observed in Calcarea and Cnidaria.

The order Haplosclerida was not resolved as monophyletic, since Suborder Spongillina (freshwater sponges) fell into other distantly related demosponge clades, rather than into marine Haplosclerida. The two suborders, Haplosclerina (families Callyspongiidae, Chalinidae and Niphatidae) and Petrosina (represented here by the families Petrosiidae and Phloeodictyidae) were not supported as monophyla (Fig. 6). These results are congruent with results from former analyses of SSU rRNA, 28S rRNA and cytochrome oxidase subunit I [20,22,52].

According to our analysis, *Amphimedon queenslandica *(Family Niphatidae) is most closely related to *Oceanapia *sp. (Family Phloeodictyidae), with *Xestospongia muta *(Family Petrosiidae) as sister taxon, and both nodes in the tree are very highly supported by posterior probability (PP) values in both Bayesian analyses. Other species of the family Niphatidae (*Niphates *sp. and *Dasychalina fragilis*) are not closely related to each other or to *Amphimedon queenslandica *(Fig. 6). In addition, other members of families Petrosiidae and Phloeodictyidae are not found in a closer relationship to the three species clade. Our results were mostly concordant with Redmond et al. [41], but with higher support values in several clades. We did not find any monophyletic haplosclerid families or genera in our taxon set. Differences between our results and the previous study are highlighted at the nodes in Fig. 5. We could not recover monophyletic Petrosiidae in clade I, and relationships of several clade III taxa differed. Furthermore, *Xestospongia muta *and *Oceanapia *sp. cluster in one clade (IV) (including *Amphimedon queenslandica*).

### Successive evolution of additional helices in marine haplosclerids

Within haplosclerids, the evolution of additional helices can be reconstructed by plotting structures to the well-supported phylogenetic backbone (Fig. 6). Primary sequences of these motifs were not included in the tree construction (Fig. 5) due to ambiguous alignment, but can be regarded as additional phylogenetic characters. The helical insertions apparently evolved in at least two steps, which fits the findings of the SSU rRNA gene tree strikingly well. The relationships within marine haplosclerids can be described as four well-supported (PP > 0.97) nested clades I-IV that display different stages of secondary structure evolution (see above and Figs. 5, 6). Clade I contains all marine Haplosclerida. The basal diverging taxa lack any large insertions that are typical for other marine Haplosclerida. However, the predicted structure within helix 43 differs from the standard structure in this region found in other Porifera (compare outgroup in Fig. 6) and displays a larger bulge of unpaired bases at the insertion point of the larger helical structures found within all taxa in Clade II. This bulge may be the precursor for the extensions at this position observed in Clade II. Within basal diverging taxa of Clade II (i.e. Clade II without Clade III), a similar bulge is found for *Xestospongia muta *in helix E23_14 at the insertion-site of subsequent extensions in Clade III, but not the other sequences lacking E23_14.1.

Larger insertions appeared in helices E23_1 and 43 'simultaneously' (according to phylogenetic resolution recovered by our analyses) in the common ancestor of Clade II taxa. The three taxa of Clade IV according to our minimum free energy calculations share an additional helix 43b.2 as synapomorphy (Figs. 4, 5, 6).

After the introduction of helical insertions in helices E23_1 and 43, a long extension evolved within helix E23_14 as a synapomorphy in Clade III. An autapomorphy for *Dasychalina fragilis *is an additional helix formed b insertions within E23_1. Within the complete taxon of marine haplosclerids, no loss of formerly gained additional helical insertions has been documented, therefore, no SSU rRNA molecule from a descendent of a taxon with extraordinary features has returned to the ancestral basic metazoan core structure.

## Discussion

### Unusual patterns within poriferan SSU rRNA secondary structure

We reported the secondary structures of a variety of poriferan SSU rRNA sequences, and suggest structure predictions for secondary structure motifs that are specific for some lineages, i.e. marine Haplosclerida (= Haplosclerida with the exception of members of the Spongillina) and Hexactinellida. Such additional helical insertions occur in a variety of eukaryotes and are known to be homoplasies, because they occur in several, not closely related taxa [50]. Our data shows that such structures are also present in early diverging Metazoa (sponges).

Insertions in helix E23_1 evolved independently in Hexactinellida and the marine Haplosclerida (Clade II), which is evident (a) from our phylogenetic analyses that captured 'snapshots' of the evolution of helices within the marine Haplosclerida (Fig. 6) and (b) from the observations that insertions appear at different positions within Helix E23_1 (compare Fig. 3 with Fig. 4). Although additional helical insertions are present within the E23-extension fragment in various eukaryotic taxa, to our knowledge, none have been reported within helix E23_14, which is therefore synapomorphic for Clade III haplosclerids. Interestingly, helical insertions within haplosclerids first appeared in the typical regions for such insertions, namely within helices E23_1 and 43, before they evolved within E23_14 (see Fig. 6). Therefore, the evolution of extensions at more common insertion sites might be a prerequisite for the evolution of additional helical structures within E23_14.

Higher Metazoa with unusual SSU rRNA structures also contain unusual motifs in their large ribosomal subunit (LSU, 28S), e.g., in branchiopod Crustacea [53]. In sponges, additional motifs in a fragment of the LSU have previously been reported for Hexactinellida and marine haplosclerid demosponges [52], but not for non-haplosclerid demosponges or Calcarea. This is in striking accordance with our SSU rRNA findings, and encourages further studies of the complete LSU secondary structure of these taxa. Since both rRNA units are encoded in one translational unit, the same mechanisms may be responsible for the formation of extra helical features in both rRNA molecules.

Remarkably, not only the nuclear rRNAs display unusual secondary structure motifs in marine Haplosclerida. In the recently published mitochondrial genome of the haplosclerid *Amphimedon queenslandica*, both of the mitochondrial (mt) rRNA genes (12S and 16SrRNA) also contain additional helices that are not found in other demosponges [54]. Although this may be a coincidental observation and needs to be verified by data from additional haplosclerid mt rRNA sequences, it is possible that the same selection mechanisms act on the nuclear and mt rRNA in this taxon. However, such correlations do not exist in all taxa, since the recently sequenced mitochondrial genomes of Hexactinellida [55] contain extremely short rRNAs (compared to the one found in Demospongiae), in contrast to the large insertions in the hexactinellid nuclear rRNAs (e.g., see Fig. 3).

For the nuclear SSU rRNA, the fact that extra-helical structures are found in the E23-extension region and helix 43 in various taxa indicates that these regions are under less functional constraints than are the core regions of SSU rRNA; Wuyts et al. [50] showed by considering the tertiary structure of rRNA that nucleotide variability increases with distance from the ribosome centre. Eukaryotic insertion sites for additional helices are therefore located in the same, or similar regions at the (3D-) periphery of rRNA molecules. The authors concluded that these insertions do not interfere with the ribosomal function of the ribosome and can therefore arise independently within different lineages, similar to our observations in Hexactinellida and marine haplosclerids.

### Phylogenetic value of rRNA features

We demonstrated different applications of SSU rRNA features for phylogenetic analyses:

#### Base composition and synapomorphic base exchanges

Base compositions of SSU rRNA differ strikingly between Demospongiae and Calcarea. The GC contents of the (much more diverse) Demospongiae are always higher and show a wider range of variability than the ones of Calcarea. For Hexactinellida, only five sequences were available (of which one is probably a non-functional copy), therefore general conclusions regarding their GC contents should be interpreted with care. However, for the few sequences available the GC contents fell into the ranges observed in Calcarea and Demospongiae.

Several apomorphic positions identified in calcarean SSU rRNA allow to unambiguously distinguish between the two subclasses Calcinea and Calcaronea, thus supporting other morphological and molecular data [13,19,56,57].

#### RNA models for phylogeny estimation and evolution of additional helical structures as evolutionary markers

The Bayesian phylogenetic reconstructions using structure-defined partitions with different rRNA models for doublets in MrBayes 3.1.2 and PHASE 2 yielded very similar tree topologies with increased support for several nodes compared to the Maximum Parsimony (MP) and Maximum Likelihood (ML) analyses presented by Redmond et al. [41] (Fig. 5). Partitioned analyses using rRNA models as applied in our analysis, have been reported to result in better-supported topologies (for sponges: [13,14]). However, other factors may have contributed to our findings, e.g., it is known that Bayesian posterior probability values are often higher than corresponding nonparametric bootstrap values and may even provide support for the 'wrong' clades in studies with simulated data [58]. The relevant important haplosclerid clades were supported with very high PP values (> 97%). These high values should overcome eventual problems of support overestimation. Also, support for 'wrong' clades is unlikely to be a problem in our results, since the topologies from Redmond et al. [41] are mostly concordant with ours. Regarding the general differences in bootstrap and PP values, the different software packages used and the difference in the data set (taxon sampling, alignment and included sites) may have contributed to the higher support found for several clades.

Although standard models of nucleotide evolution violate the assumption of independent evolution of all sites when also applied to paired sites as done by Redmond et al. [41], this seems to have little impact on nodes with high support values in the case of the demosponge dataset studied (compare Fig. 1 in [41] with our Fig. 5). This suggests that the biases introduced by the use of less well fitting 'standard' rRNA models may have a higher impact on clades that are difficult to resolve (e.g., due to noisy data), whereas a strong phylogenetic signal will be recovered even if a sub-optimal evolutionary model is used for analyses.

*Amphimedon queenslandica*, the target of the Sponge Genome Sequencing project, did not cluster with any other representative of its Family Niphatidae. Likewise, neither the other haplosclerid families (Callyspongiidae, Chalinidae, Petrosiidae, Phloeodictyidae), nor the genera (*Callyspongia*, *Haliclona*, *Petrosia*) could be recovered as monophyletic, (besides of the genus *Petrosia*) in accordance with the SSU-rRNA based findings of Redmond et al. [41]. Strikingly, these inferred relationships are supported by the presence or absence of secondary structure motifs within Haplosclerida: different members of the families Niphatidae, Petrosiidae and Phloeodictyidae show a different number of specific insertions that are congruent with the phylogenetic relationships that we previously inferred without the inclusion of these extended regions (see Figs. 5, 6). The presence and absence of such helices are therefore good phylogenetic indicators for these relatively closely related taxa, even though alignment of the primary sequence of these helices (and cladistic or phenetic sequence analysis) between all taxa is difficult due to high evolutionary rates. Homology inference of sites in hypervariable regions according to their secondary structure is known to be problematic. Functional constraints are probably more relaxed in these regions, and the observed evolution of insertions is driven by unknown mutational mechanisms, which might tend to produce similar motifs by homoplasy [59]. In contrast, within marine haplosclerids, no loss of helical insertions that arose at some earlier point of their evolutionary history occurred (Fig. 6). Furthermore, no independent homoplasic helical insertions ever appeared in the same positions within SSU rRNA. Considering our findings, the presence and absence of large helical insertions appears to provide strong phylogenetic information at selected taxonomical levels.

A similar phylogenetic information value of additional helical structures may be present for insertions in Helix E23_1 of Hexactinellida, although generalized conclusions are limited by the small sample size. Nonetheless, the insertions in Hexasterophora (*Acanthascus*, *Oopsacas*, *Aphrocallistes*, and *Farrea*) are predicted to possess two additional helices, while the insertion found in the only considered amphidiscophoran, *Semperella schulzei*, forms only one helix (Fig. 3). It is evident that integration of secondary structure information in sequence alignment and analyses (in the form of rRNA substitution models) will optimize rRNA phylogenies considerably.

## Conclusion

The SSU rRNA provides far more valuable phylogenetic information than just its primary sequence. Even simple features like base composition already bear enough information to distinguish between the two higher sponge taxa Calcarea and Demospongiae (Fig. 1). Unusual secondary structures can lend further support to results from independent phylogenetic inferences, as we showed for helical insertions in marine haplosclerid demosponges (Fig. 6) and for a small number of hexactinellid sponges (Fig. 3). In this way, otherwise neglected hypervariable insertions can yield further support to a given topology. Although we only explored additional structures of SSU rRNA in sponges, our results should encourage further studies. Especially the study of LSU rRNA structure seems promising in this regard, since this gene is more variable than SSU rRNA and contains large extension regions that strongly differ among higher taxa [60]. On the intraspecific level, the even more variable internal transcribed spacer regions (ITS 1 and ITS 2) can provide secondary structure features of phylogenetic value (e.g. [61]). Relating secondary structure to sequence information will allow the phylogenetic signal of the huge numbers of rRNA sequences currently available in Genbank to be considerably increased. Our newly generated database of Porifera SSU rRNA secondary structures will facilitate the inclusion of secondary structure information in phylogenetic analyses.

## Authors' contributions

OV contributed to the conception and design of the study, acquired, analyzed (including development of analytical Perl scripts) and interpreted the data, and wrote the manuscript. DE and GW contributed to the conception and design of the study, and critical revision of the manuscript. All authors read and approved the final version of the manuscript.

## Supplementary Material

Additional file 1 Taxonomy and GenBank accession numbers of downloaded poriferan SSU rRNA sequences. Click here for file

Additional file 2 Trace IDs from GenBank trace archive used to assemble the *Amphimedon queenslandica *SSU rDNA sequence. Click here for file

Additional file 3 Compensatory base changes and alignments of predicted secondary structures of clade III marine Haplosclerida (Demospongiae) and Hexasterophora (Hexactinellida). Click here for file

Additional file 4 Base composition and fragment lengths of examined poriferan SSU rRNA fragment (see Methods and Fig. 1). Click here for file

Additional file 5 Alignment with secondary structure information and character set. Secondary structure is provided for regions that are included in the phylogenetic analyses only. Additional secondary structure information is available from our poriferan SSU rRNA database. Click here for file
