# Supplementary material for: Molecular evolution of rDNA in early diverging Metazoa: First comparative analysis and phylogenetic application of complete SSU rRNA secondary structures in Porifera
Source: BMC Evol Biol. 2008 Feb 27;8:69. doi: 10.1186/1471-2148-8-69 (PMC2289807; doi:10.1186/1471-2148-8-69)
Supplement: Additional file 3 — Compensatory base changes and alignments of predicted secondary structures of clade III marine Haplosclerida (Demospongiae) and Hexasterophora (Hexactinellida). [file 1471-2148-8-69-S3.pdf]

# Compensatory base changes in additional helices

In the presented structure plots, positions with base changes supporting the structure are encircled. Positions with mismatches in some sequences are in grey. In the tables, pairs at positions with compensatory base changes are in dark green, semi-compensatory change in light green. Mismatches are shaded in orange. Numbers of occurrence are given after each pair. Only sequences, which allowed an alignment, were included.

## Marine Haplosclerida

.

### Helix E23_1a.1

14 taxa, *Cribochalina* was aligned according to minimum free energy folding predictions (pos.1 is included in E23_1b and not shown in structure)

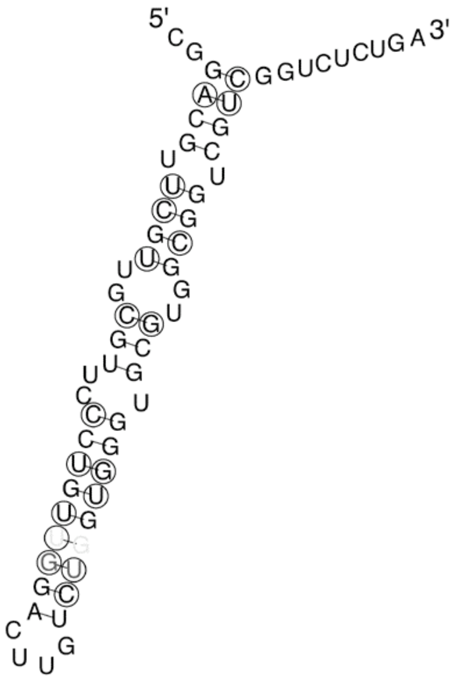


| 5' | 3' | | | | | | |
|---|---|---|---|---|---|---|---|
| 4 | 60 | G:C 13 | G:U 1 | | | | |
| 5 | 59 | A:U 9 | G:U 4 | G:C 1 | | | |
| 6 | 58 | C:G 14 | | | | | |
| 7 | 57 | G:C 13 | G:U 1 | | | | |
| 9 | 55 | U:G 11 | C:G 3 | | | | |
| 10 | 54 | C:G 11 | U:G 2 | G:C 1 | | | |
| 11 | 53 | G:C 11 | G:U 2 | -:- 1 | | | |
| 12 | 52 | U:G 11 | U:A 1 | C:G 1 | | | |
| 15 | 49 | C:G 13 | U:A 1 | | | | |
| 16 | 48 | G:C 13 | G:U 1 | | | | |
| 17 | 47 | G:U 12 | A:U 1 | U:U 1 | | | |
| 20 | 45 | C:G 12 | U:G 2 | | | | |
| 21 | 44 | C:G 14 | | | | | |
| 22 | 43 | U:G 6 | C:G 4 | U:A 3 | G:C 1 | | |
| 23 | 42 | G:U 7 | G:C 6 | U:G 1 | | | |
| 24 | 39 | C:G 7 | U:G 6 | U:U 1 | | | |
| 25 | 38 | U:G 7 | C:G 3 | U:U 3 | C:A 1 | | |
| 27 | 36 | G:C 5 | G:U 4 | C:G 2 | U:G 1 | A:U 1 | C:U 1 |
| 28 | 35 | G:C 12 | G:U 1 | U:A 1 | | | |
| 29 | 34 | A:U 12 | G:C 1 | -:- 1 | | | |

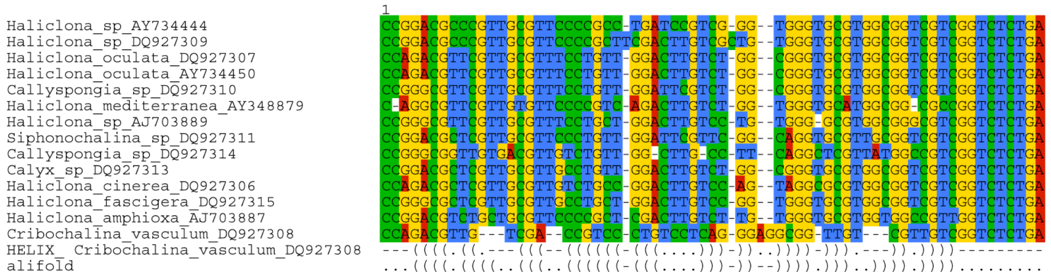

# Helix E23_14a.1

11 taxa (right structure) +2 taxa (left structure). Alignment and compensatory base changes are combined.

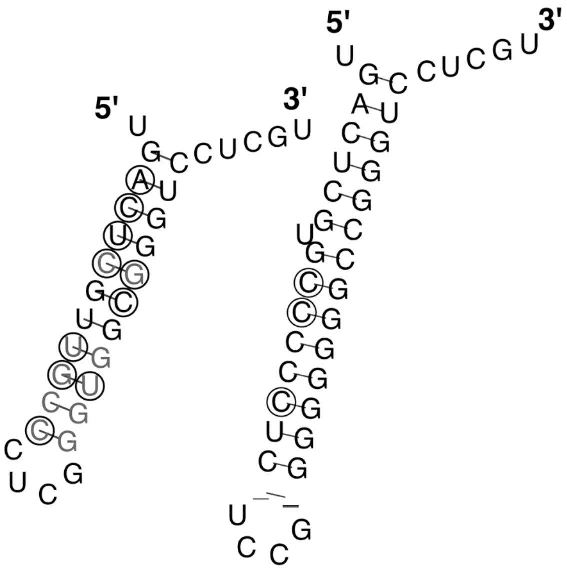


| 5' | 3' | | | | | |
|---|---|---|---|---|---|---|
| 2 | 36 | G:C 13 | | | | |
| 3 | 35 | A:U 11 | G:U 2 | | | |
| 4 | 34 | C:G 9 | U:G 4 | | | |
| 5 | 33 | U:G 8 | C:G 5 | | | |
| 6 | 32 | C:G 10 | U:A 1 | U:G 1 | C:A 1 | |
| 7 | 31 | G:C 11 | G:U 2 | | | |
| 9 | 30 | U:G 11 | G:C 2 | | | |
| 10 | 29 | U:G 8 | C:G 4 | -:- 1 | | |
| 11 | 28 | G:U 7 | G:C 2 | A:U 1 | C:G 1 | U:G 1 | -:- 1 |
| 12 | 27 | C:G 12 | -:- 1 | | | |
| 13 | 26 | C:G 7 | U:G 5 | -:- 1 | | |
| 14 | 25 | -:- 11 | C:G 1 | U:G 1 | | |
| 15 | 24 | -:- 11 | U:G 2 | | | |
| 16 | 23 | -:- 11 | C:G 2 | | | |
| 17 | 22 | -:- 12 | C:G 1 | | | |

```
                              1
Haliclona_sp_AY734444         TGACTCGTGTCCCTTCCTTCGGGGGGGGGCCGGGTCCTCGT
Haliclona_sp_DQ927309         TGACTCGTGCTCCCTC-TCCG-GGGGGGGCCGGGTCCTCGT
Haliclona_oculata_DQ927307    TGACTCG-TTGCT----CTCG----GGTGGCGGGTCCTCGT
Haliclona_oculata_AY734450    TGACTCG-TTGCT----CTCG----GGTGGCGGGTCCTCGT
Callyspongia_sp_DQ927310      TGACTCG-TTGCC----TTCG----GGTGGCGGGTCCTCGT
Callyspongia_sp_DQ927314      TGGCCCG-TCACT----CTCG----GGTGGTGGGTCCTTGC
Haliclona_mediterranea_AY348879 TGATCCG-TTGCC----TTCG----GGTGGCAGGTCCTCGT
Haliclona_sp_AJ703889         TGATTCG-TTGCC----TTCG----GGTGGCGGGTCCTCGT
Siphonochalina_sp_DQ927311    TGATCCG-TCGCT----CTCG----GGCGGTGGGTCCTCGT
Calyx_sp_DQ927313             TGATCTG-TCGCT----CTCG----GGCGGCGGGTCCTCGT
Haliclona_cinerea_DQ927306    TGGCCTG-T--------TTCG--------GCAGGTCCTCGT
Haliclona_fascigera_DQ927315  TGACTCG-TTGCC----CTCG----GGTGGCGGGTCCTCGT
Haliclona_amphioxa_AJ703887   CGACTCG-TTGCC----CTCG----GGTGGCGGGTCCTCGT
alifold                       .((((((.(((((((((....))))))))))))))).....
```

# Helix 43b.1

13 taxa, parts of conserved helix 43 (a+b) included in structure and alignment. Compensatory base changes are only shown for insertion.

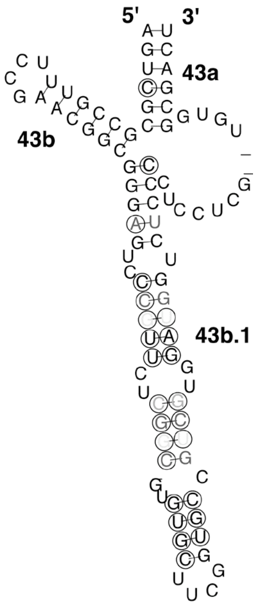


| 5' | 3' | | | | | | | | |
|---|---|---|---|---|---|---|---|---|---|
| 23 | 73 | G:C 12 | G:U 1 | | | | | | |
| 24 | 72 | G:C 13 | | | | | | | |
| 25 | 71 | G:C 13 | | | | | | | |
| 26 | 70 | A:U 10 | G:U 2 | A:A 1 | | | | | |
| 27 | 69 | G:C 13 | | | | | | | |
| 30 | 67 | C:G 12 | U:G 1 | | | | | | |
| 31 | 66 | C:G 11 | U:G 1 | G:G 1 | | | | | |
| 32 | 65 | G:C 4 | G:U 2 | A:U 3 | U:U 1 | C:C 1 | C:U 2 | | |
| 33 | 64 | U:A 10 | U:G 2 | A:U 1 | | | | | |
| 34 | 63 | U:G 10 | C:G 2 | G:C 1 | | | | | |
| 37 | 60 | C:G 7 | G:C 2 | U:G 1 | U:A 1 | G:A 1 | C:U 1 | | |
| 38 | 59 | G:C 9 | G:U 2 | A:U 1 | U:U 1 | | | | |
| 39 | 58 | G:U 3 | A:U 3 | U:G 2 | G:C 1 | U:A 1 | A:G 1 | C:A 1 | G:G 1 |
| 40 | 57 | C:G 10 | U:G 2 | U:U 1 | | | | | |
| 44 | 55 | G:C 12 | C:G 1 | | | | | | |
| 45 | 54 | U:G 9 | C:G 3 | U:A 1 | | | | | |
| 46 | 53 | G:U 7 | G:C 4 | A:U 2 | | | | | |
| 47 | 52 | C:G 9 | U:G 4 | | | | | | |

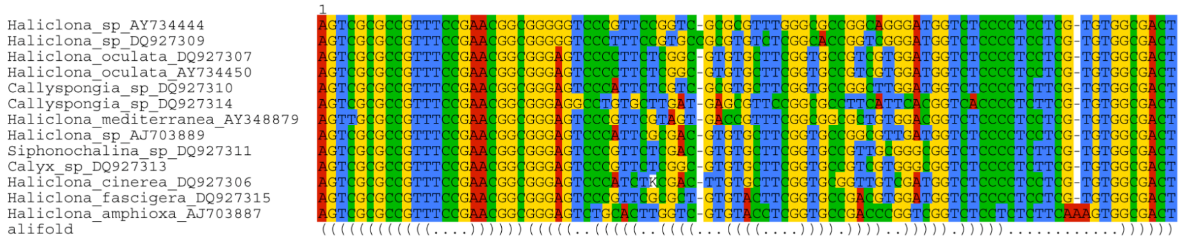


# Comparison of structures inferred comparatively (considering compensatory changes) and structures inferred only with minimum free folding predictions for *Haliclona* sp. (AY734444).

Arrows show differences.

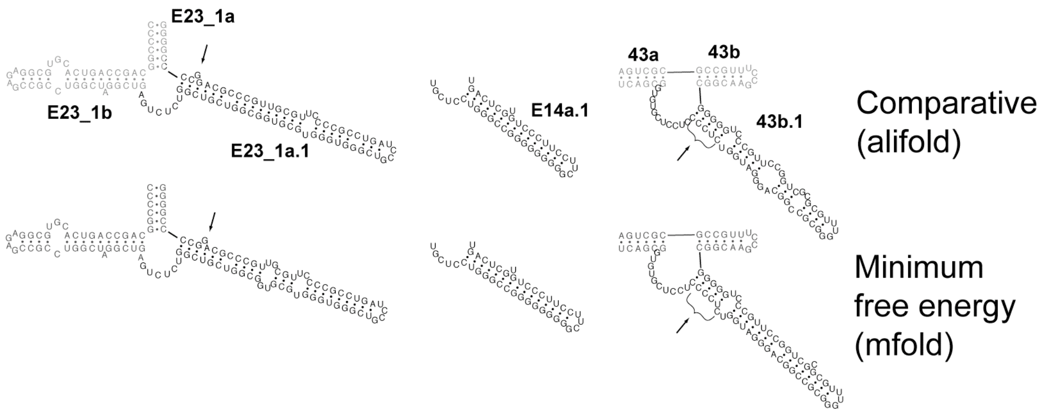

# Hexasterophora (Hexactinellida)

## Helix E23_1b.1 and E23_1b.2

Initial alignments were refined and combined after separated alifold analyses of Hexactinosida (*Aphrocallistes* +*Farrea*) and Lyssacinosida (*Acanthascus*+*Oopsacas*)

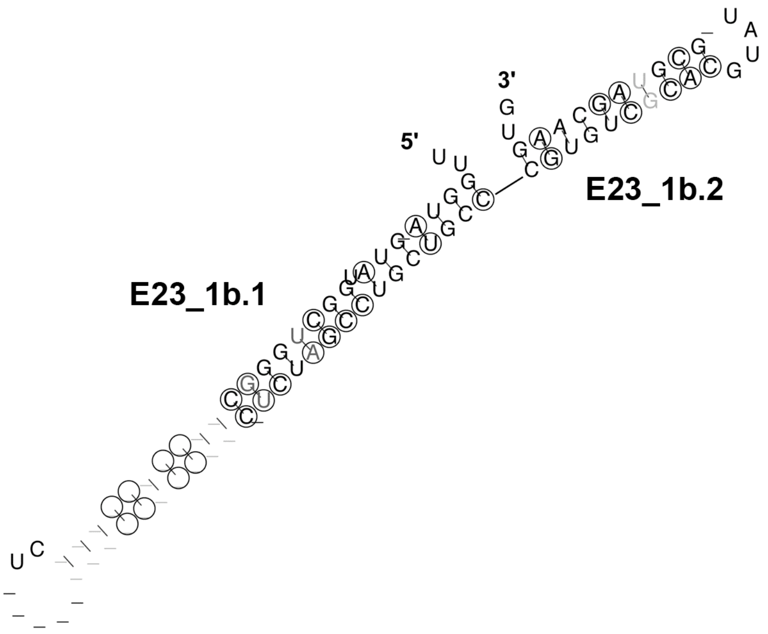


| 5' | 3' | | | | | | | |
|---|---|---|---|---|---|---|---|---|
| 3 | 62 | G:C | 3 | G:U | 1 | | | Helix E23_1b.1 |
| 4 | 61 | G:C | 4 | | | | | |
| 5 | 60 | U:G | 4 | | | | | |
| 6 | 59 | A:U | 2 | G:C | 1 | G:U | 1 | |
| 8 | 58 | G:C | 4 | | | | | |
| 9 | 57 | U:G | 4 | | | | | |
| 10 | 56 | A:U | 3 | G:U | 1 | | | |
| 12 | 55 | G:C | 2 | G:U | 2 | | | |
| 13 | 54 | G:C | 2 | G:U | 2 | | | |
| 14 | 53 | C:G | 2 | U:A | 1 | U:G | 1 | |
| 15 | 52 | U:G | 2 | U:A | 1 | -:A | 1 | |
| 16 | 51 | G:U | 4 | | | | | |
| 17 | 50 | G:C | 3 | G:U | 1 | | | |
| 18 | 49 | G:U | 2 | U:G | 2 | U:U | 1 | |
| 19 | 47 | G:C | 2 | C:G | 2 | | | |
| 20 | 46 | U:G | 2 | -:- | 2 | | | |
| 21 | 45 | G:U | 2 | -:- | 2 | | | |
| 22 | 44 | G:C | 1 | U:A | 1 | -:- | 2 | |
| 23 | 43 | G:C | 1 | U:G | 1 | -:- | 2 | |
| 24 | 42 | U:G | 2 | -:- | 2 | | | |
| 25 | 41 | U:A | 1 | G:U | 1 | -:- | 2 | |
| 26 | 40 | C:G | 1 | G:U | 1 | -:- | 2 | |
| 27 | 39 | C:G | 1 | G:G | 1 | -:- | 2 | |
| 28 | 38 | C:G | 2 | -:- | 2 | | | |
| 29 | 37 | U:G | 2 | -:- | 2 | | | |
| 63 | 87 | C:G | 4 | | | | | Helix E23_1b.2 |
| 64 | 86 | U:A | 2 | G:U | 2 | | | |
| 65 | 85 | U:A | 4 | | | | | |
| 66 | 84 | G:C | 4 | | | | | |
| 67 | 83 | U:A | 1 | U:G | 3 | | | |
| 68 | 82 | C:G | 2 | U:A | 2 | | | |
| 69 | 81 | G:U | 2 | R:U | 1 | G:Y | 1 | |
| 70 | 80 | C:G | 2 | U:G | 2 | | | |
| 71 | 79 | G:C | 2 | A:U | 2 | | | |
| 72 | 78 | C:G | 2 | U:G | 2 | | | |

```
                             1
Oopsacas_minuta_AF207844     UUGGUG-GUAUGGC-GGGGUGGUUGCCCUGAGA---GGGGUGGCUGCCUUUAGCUUGCUGCUCUUGUCGUGUGUAUUGCGUGACAAGUG
Acanthascus_dawsoni_AF100949 UUGGUG-GUGUGGCUGGGGUGUGUUGCCUUCAC---GGGUAGCAUGC-UCUGGUUUGCCGCCCUUGUCRUGUGUAUUGCGUGGCAAGUG
Farrea_occa_AF159623         UUGGUAUGUA-GGUUGGUC----------CUUGUGU----------G-GCUGAUCUGCUGCCCGUGUUGCACGUGU-GUGUAGCAUGUG
Aphrocallistes               UUGGUAUGUAUGGUUGGUC----------CU--UGU----------G-UCUAGCCUGCUGCCCGUGUUGCACGUGU-GUGYAGCAUGUG
alifold                      ..((((.(((.(((((((((((((((((.......)))))))))).))))))))))))))(((((((((((.....)))))))))))..
```
